# Hybrid Aspen Expressing a Carbohydrate Esterase Family 5 Acetyl Xylan Esterase Under Control of a Wood-Specific Promoter Shows Improved Saccharification

**DOI:** 10.3389/fpls.2020.00380

**Published:** 2020-04-08

**Authors:** Zhao Wang, Prashant Mohan-Anupama Pawar, Marta Derba-Maceluch, Mattias Hedenström, Sun-Li Chong, Maija Tenkanen, Leif J. Jönsson, Ewa J. Mellerowicz

**Affiliations:** ^1^Department of Chemistry, KBC Chemical-Biological Centre, Umeå University, Umeå, Sweden; ^2^Department of Forest Genetics and Plant Physiology, Swedish University of Agricultural Sciences, Umeå, Sweden; ^3^Department of Food and Environmental Sciences, University of Helsinki, Helsinki, Finland

**Keywords:** acetyl xylan esterase, hybrid aspen, *Populus*, xylan, acetyl, enzymatic saccharification

## Abstract

Fast-growing broad-leaf tree species can serve as feedstocks for production of bio-based chemicals and fuels through biochemical conversion of wood to monosaccharides. This conversion is hampered by the xylan acetylation pattern. To reduce xylan acetylation in the wood, the *Hypocrea jecorina* acetyl xylan esterase (*Hj*AXE) from carbohydrate esterase (CE) family 5 was expressed in hybrid aspen under the control of the wood-specific *Pt*GT43B promoter and targeted to the secretory pathway. The enzyme was predicted to deacetylate polymeric xylan in the vicinity of cellulose due to the presence of a cellulose-binding module. Cell-wall-bound protein fractions from developing wood of transgenic plants were capable of releasing acetyl from finely ground wood powder, indicative of active AXE present in cell walls of these plants, whereas no such activity was detected in wild-type plants. The transgenic lines grew in height and diameter as well as wild-type trees, whereas their internodes were slightly shorter, indicating higher leaf production. The average acetyl content in the wood of these lines was reduced by 13%, mainly due to reductions in di-acetylated xylose units, and in C-2 and C-3 mono-acetylated xylose units. Analysis of soluble cell wall polysaccharides revealed a 4% reduction in the fraction of xylose units and an 18% increase in the fraction of glucose units, whereas the contents of cellulose and lignin were not affected. Enzymatic saccharification of wood from transgenic plants resulted in 27% higher glucose yield than for wild-type plants. Brunauer–Emmett–Teller (BET) analysis and Simons’ staining pointed toward larger surface area and improved cellulose accessibility for wood from transgenic plants compared to wood from wild-type plants, which could be achieved by *Hj*AXE deacetylating xylan bound to cellulose. The results show that CE5 family can serve as a source of enzymes for *in planta* reduction of recalcitrance to saccharification.

## Introduction

Bioconversion of woody biomass has potential to provide advanced biofuels and bio-based materials. However, wood is relatively resistant to chemical and biological conversion processes, which necessitates costly processing and reduces recovery of desired products (Himmel et al., 2007; Zhao et al., 2012; McCann and Carpita, 2015). The main constituents of this biomass, i.e., lignin, hemicelluloses, and cellulose, and their interactions in the cell wall, all contribute to the complex structure of wood and its recalcitrance (Ragauskas, 2013).

The importance of acetylation for the recalcitrance of hemicelluloses received attention relatively long time ago (Biely et al., 1985; Grohmann et al., 1989). In woody biomass from hardwoods, most acetyl groups are on Xyl*p* (xylopyranosyl) units of xylan. The fraction of acetyl groups in hardwoods varies from around 3.5 to 4.4% (w/w) on dry-weight basis (Pawar et al., 2013), where 40–70% of the Xyl*p* units can be acetylated at the C-2 and/or C-3 positions of Xyl*p* units (Teleman et al., 2000, 2002; Kabel et al., 2003; Goncalves et al., 2007). Distribution of acetylated Xyl*p* units along the xylan chain of woody dicots is regulated by the activity of acetyl transferase ESK1/TBL29 (Grantham et al., 2017). Typically, every second unit is mono- or di-acetylated, and this pattern enables the xylan backbone to interact with the hydrophilic face of cellulose microfibrils in two-fold screw conformation (Busse-Wicher et al., 2014; Chong et al., 2014; Grantham et al., 2017).

Acetylation on xylan chains may contribute to biomass recalcitrance by changing surface hydrophobicity and thereby inhibiting productive binding of hydrolytic enzymes, and by causing steric hindrance of enzymes targeting cellulose and xylan (Pan et al., 2006; Busse-Wicher et al., 2014). Acetyl groups are hydrolyzed by pretreatment of lignocellulosic biomass yielding acetic acid, which is, however, a quantitatively significant inhibitor of microbial fermentation processes (Jönsson and Martin, 2016). Reduced acetylation of plant cell walls would therefore increase the accessibility of polysaccharides and reduce the inhibition of fermenting microbes.

Strong reduction of acetylation, especially in xylan, obtained by knocking out components of a xylan acetylation machinery typically causes dwarfism, reduced mechanical strength of the stem, collapsed vessels, and stunted plant growth (Lee et al., 2011b; Manabe et al., 2013; Yuan et al., 2013, 2016). Plants with severely reduced acetylation may therefore not necessarily exhibit increased sugar yield after enzymatic saccharification (Lee et al., 2011b; Xiong et al., 2013; Yuan et al., 2013). However, moderate decrease of xylan acetylation in hybrid aspen was not only well-supported by plants but also lead to better saccharification (Pawar et al., 2017b). On the other hand, excess xylan acetylation in rice, while providing some beneficial effects on saccharification, disrupted the structure of the secondary cell wall and lead to growth defects (Zhang et al., 2017).

Microbial enzymes with acetyl xylan esterase (AXE) activity could be used *in planta* to reduce xylan acetylation. These enzymes are grouped in at least eight Carbohydrate Esterase (CE) families that differ with regard to protein structure and other properties (Biely, 2012; Pawar et al., 2013). Previous studies have shown that introduction of an *Aspergillus niger* AXE1 (*An*AXE1) from CE1 in *Arabidopsis* or in hybrid aspen and targeting the enzyme to the cell wall for post-synthetic xylan deacetylation significantly improved the cellulose digestibility without changing the growth properties of these plants (Pawar et al., 2016, 2017a). Post-synthetic xylan deacetylation was considered as a more promising strategy than synthetic xylan deacetylation in the Golgi, since the latter could induce excess glucuronidation (Donev et al., 2018) caused by the promiscuous activity of glucuronyl transferases GUX1 and GUX2 (Grantham et al., 2017). These results encourage further trials with microbial enzymes capable of deacetylation of xylan in cell walls.

Here we are testing the AXE from the filamentous fungus *Hypocrea jecorina* (formerly *Trichoderma reesei*), *Hj*AXE, from family CE5. Compared to CE1 AXEs, which have broad specificity to different poly- and oligosaccharides, the AXEs from CE5 are thought to be more specific to polymeric xylan (Biely et al., 2011). Subtle differences were observed between CE1 and CE5 AXEs *in vitro* when deacetylating different acetylated xylo-oligosaccharides (Koutaniemi et al., 2013). Moreover, unlike CE1 AXEs, CE5 *Hj*AXE has a C-terminal cellulose binding domain (Margolles-Clark et al., 1996). These features expectedly would affect the performance of the members of these families when expressed *in planta*. A pairwise alignment of *An*AXE1 and *Hj*AXE (data not shown) indicated that the amino-acid sequence identity was < 20%, which further accentuates the difference between CE1 and CE5 enzymes.

We found that transgenic hybrid aspen expressing *Hj*AXE has normal growth in the greenhouse whereas its xylan is deacetylated by approximately 13% compared to the wild type (WT). The wood of such plants had improved bioprocessing properties along with increased cellulose accessibility. These results support the suitability of CE5 AXEs for post-synthetic xylan deacetylation.

## Materials and Methods

### Plant Material

Transgenic hybrid aspen (*Populus tremula* L. × *tremuloides* Michx.) lines were generated as described previously (Ratke et al., 2015). The lines harbored the codon-optimized cDNA of *Hypocrea jecorina AXE* (*HjAXE*), GenBank accession CAA93247 (Margolles-Clark et al., 1996) cloned behind the wood-specific promoter in *pK-GT43B-GW7* (Ratke et al., 2015), and were denoted as WP:CE5. Twenty independent transgenic lines were screened *in vitro* for expression of *Hj*AXE, and the 11 best lines were further screened in the greenhouse. The three most highly expressing lines were finally selected and grown in the greenhouse along with WT control for 8 weeks. The growth conditions were as follows: light photoperiods 18 h [using HQI-TS 400W/DH metal halogen lamps (Osram, Munich, Germany) to supplement daylight when necessary], 20/15°C (day/night) temperatures, and 60–70% relative humidity. The plants were watered daily, fertilized once per week with Rika-S (Weibulls Horto, Hammenhög, Sweden) and shifted weekly to avoid any position effects. Stem height was periodically measured, and the average internode length for internodes 19–35, and the stem diameter for internodes 20 and 40 were determined at the time of harvest.

### Transcript Level

Total RNA was extracted from developing xylem tissue of hybrid aspen by using the Cetyl Trimethyl Ammonium Bromide (CTAB) extraction method (Chang et al., 1993). The cDNA was synthesized from 1 μg of RNA using a cDNA biosynthesis Bio-Rad kit (Bio-Rad Laboratories AB, Sundbyberg, Sweden). Diluted cDNA (20–30 times) was used for transcript analysis of transgenic plants. The expression was normalized to ubiquitin (Potri.005G198700) and tubulin (Potri.001G464400), and presented relative to the levels in the lowest-expressing line as previously described (Pawar et al., 2017a). The primers of reference and target genes are provided in Supplementary Table S1.

### Acetyl Esterase Activity Assay

Soluble and wall-bound fractions of proteins were isolated from the developing wood of transgenic and WT hybrid aspen using the method described by Biswal et al. (2014) and tested for acetyl esterase activity using naturally acetylated aspen wood components as esterase substrates (Margolles-Clark et al., 1996). Aspen wood powder (particle size < 50 μm) was suspended in 50 mM sodium citrate buffer (pH 5.0) at 20 g/L and 2 μL of this suspension was incubated with 10 μg of total protein extract in a total volume of 400 μL (in the same buffer) for 24 h at 45°C. After reaction, the mixtures were denatured for 5 min at 100°C, centrifuged briefly and 10 μL of the supernatants were analyzed for the content of acetic acid by using a K-ACET kit (Megazyme, Bray, Ireland). Reaction mixtures containing denatured (10 min at 100°C) instead of fresh protein were used as negative controls. Results are presented as μmol of acetic acid produced from wood powder by 1 mg of protein in 1 h at 45°C. Three trees per each transgenic line and per WT were analyzed.

### Cell Wall Compositional Analysis

Wood from internodes 19–35 was freeze-dried, and then ground to a rough wood powder (particle size < 0.5 mm), which was then ball-milled to a fine wood powder as previously described (Gandla et al., 2015). The fine wood powder was analyzed by using Fourier transform infra-red (FTIR) spectroscopy and pyrolysis gas chromatography combined with mass spectrometry (Py-GC/MS) as previously described (Gandla et al., 2015; Pawar et al., 2017a). The data were analyzed by using SIMCA-P (Umetrics AB, Umeå, Sweden).

The acetyl content was determined according to Gille et al. (2011) by saponification of the fine wood powder. The released acetic acid was analyzed by using HPAEC (high-performance anion-exchange chromatography) as previously described (Wang et al., 2018).

Alcohol insoluble residue (AIR) of the fine wood powder was prepared (Pawar et al., 2017a). The AIR was used to determine Klason lignin, acid-soluble lignin (ASL), Updegraff cellulose, and trimethylsilyl (TMS) monosaccharides content of non-cellulosic polysaccharides as described by Gandla et al. (2015).

### Xylan Structure Analyses

#### NMR (Nuclear Magnetic Resonance) Spectroscopy Analysis

Acetylated xylan polymer was prepared from AIR by delignification and DMSO extraction (Pawar et al., 2017a). 2D 1H-13C HSQC was used to analyze the xylan polymer and spectra were acquired from a Bruker Avance III HD 850 MHz spectrometer as described by Pawar et al. (2017a).

#### OLIMP (OLIgosaccharide Mass Profiling) Analysis

AIR residue was heat-treated at 60°C for 1 h to deactivate acetyl xylan esterase and digested by pure GH10 endo-1,4-β-D-xylanase from *Aspergillus aculeatus* (*Aa*GH10) [kind gift from Novozymes A/S (Bagsværd, Denmark)]. The released xylo-oligosaccharides were desalted and separated into neutral and acidic fractions using a Graphitized Carbon SPE column (Thermo Scientific) (Chong et al., 2014). The mass spectra were acquired with atmospheric pressure matrix-assisted laser desorption/ionization-ion trap mass spectrometry (AP-MALDI-ITMS) as described by Chong et al. (2011).

### Pretreatment and Saccharification

The rough wood powder was sieved and the fraction with a particle size of 0.1–0.5 mm was used for pretreatment and saccharification. Reaction mixtures containing 50 mg wood (dry weight) and 1% (w/w) sulfuric acid were pretreated at 165°C for 10 min using a single-mode microwave system (Initiator Exp, Biotage, Uppsala, Sweden). Saccharification of pretreated and non-pretreated wood samples was performed by enzymatic digestion of 50 mg wood (dry weight) (or, for pretreated material, the solid residue remaining after the pretreatment of 50 mg wood) using a 1:1 (v/v) mixture of Celluclast 1.5L and Novozyme 188. The load of enzyme protein corresponded to 1 mg per 50 mg wood. The total mass of the reaction mixture was 1000 mg and the medium consisted of sodium citrate buffer (0.5 M, pH 5.2). Reaction mixtures were incubated for 72 h in 2-mL Sarstedt safe-seal micro-centrifuge tubes in an orbital shaker set at 170 rpm and 45°C. Aliquots withdrawn after 2 h were analyzed by using a glucometer (Accu-Chek Aviva, Roche Diagnostics, Risch-Rotkreuz, Switzerland), and data were used to calculate the glucose production rate (GPR). The yields of arabinose, galactose, glucose, mannose, and xylose in pretreatment liquids and in enzymatic hydrolyzates after 72 h incubation were determined using HPAEC, as previously described (Wang et al., 2018).

### Brunauer–Emmett–Teller (BET) Analysis

The surface area of non-pretreated and acid-pretreated sieved rough wood powder (0.1–0.5 mm) was analyzed with a single-point BET procedure using a TriStar 3000 analyzer (Micromeritics, Atlanta, GA, United States). A SmartPrep Degasser (Micromeritics) was utilized prior to the analysis with TriStar 3000 to remove potential adsorbed contaminants. The BET method is based on Langmuir theory and adsorption of nitrogen gas.

### Simons’ Staining

Simons’ staining estimates the accessibility of cellulosic materials to enzymes based on solute exclusion (Yu et al., 1995; Arantes and Saddler, 2011). A modified Simons’ staining assay (Chandra and Saddler, 2012) was used to analyze cellulose accessibility. The analysis was performed using the non-pretreated and acid-pretreated sieved rough wood powder (0.1–0.5 mm). Direct Blue (DB, Pontamine Fast Sky Blue 6BX) and Direct Orange (DO, Pontamine Fast Sky Orange 6RN) dyes were obtained from Pylam Products (Garden City, NY, United States).

### Statistical Analysis

JMP^®^ Pro program^1^ with analysis of variance (ANOVA) was used for data analysis. *Post hoc* Dunnett-test was used to compare individual transgenic lines with the WT and contrast test was used to compare all transgenic lines with the WT.

## Results

### Growth of CE5-Expressing Hybrid Aspen

**FIGURE 1:**
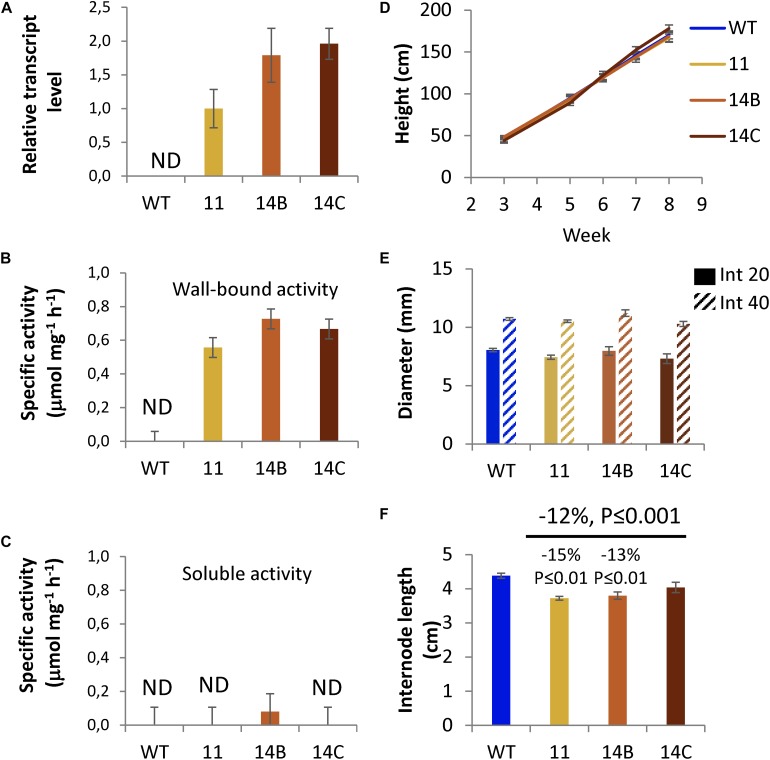
Growth and transgene expression in selected WP:CE5 lines (11, 14B, and 14C) used in this study compared to wild type (WT). **(A)** Transgene transcript levels in developing wood determined by RT-qPCR; the expression relative to two reference genes was normalized to the levels observed in line 11. AXE specific activity in wall-bound **(B)** and soluble **(C)** protein fractions isolated from developing wood, determined by release of acetic acid from acetylated aspen wood powder used as substrate. Data in **(A–C)** are means ± *SE*, *N* = 3, biological replicates. **(D)** Height growth of transgenic lines during 8 weeks in the greenhouse. **(E)** Diameter of internodes 20 and 40 at week 8. **(F)** Average internode length at week 8. Data in **(D–F)** are means ± *SE*, *N* = 6 trees per transgenic lines and 23 trees per WT. *P*-values correspond to post-ANOVA Dunnett test (for individual lines) or contrast analysis comparing all transgenic lines to WT.

Hybrid aspen lines 11, 14B, and 14C, expressing *Hj*AXE (Margolles-Clark et al., 1996) harboring the plant signal peptide of aspen cellulase *Ptxt*CEL9B3 under control of wood-specific promoter (Ratke et al., 2015), denoted WP:CE5, were grown in the greenhouse for 2 months. The three selected lines had the highest transgene expression from 20 obtained lines as described in Section “Materials and Methods.” Transgene expression in developing wood tissues was approximately two times lower in line 11 than in lines 14B and 14C (Figure 1A). Acetyl esterase activity was studied in wall-bound and soluble protein fractions using naturally acetylated aspen wood as a substrate (Margolles-Clark et al., 1996). This method was considered more specific for xylan acetyl esterases acting on polymeric xylan as compared to methods using synthetic esters as substrates. The activity was detected only in wall-bound protein fraction extracted from developing wood of WP:CE5 lines (Figure 1B). It was varying within the range of 0.56–0.73 μmol mg^–1^ protein h^–1^, consistent with the transgene expression levels. No activity was detected in wall-bound protein extracts of WT plants. No or negligible activity was recorded in the soluble protein fraction in transgenic lines and no activity was found in this fraction in the WT (Figure 1C). These data indicated that the expressed protein was active as acetyl esterase and associated with cell walls, as expected.

Plant morphology, height growth, and diameter growth of transgenic lines were similar to WT, whereas their internodes were shorter (Figures 1D–F). Since both transgenic and WT plants produced one leaf per internode, this indicates that the transgenic plants produced more leaves. Overall, the results show that the CE5 enzyme is expressed and active in the cell walls of selected transgenic lines without negatively affecting their growth or development.

### Lignin and Carbohydrates Contents

To investigate potential effects of expression of CE5 AXE on the wood cell wall chemical composition, the contents of lignin and Updegraff cellulose, the yields of trimethyl-silyl (TMS) monosaccharides, and the composition of wood pyrolyzates were analyzed (Table 1). The contents of Klason and acid-soluble lignin, and Updegraff cellulose did not show consistent differences among the transgenic lines compared to WT (Table 1). Analysis using Py-GC/MS (Table 1) showed a small increase in H lignin units (which are a minor lignin component) in transgenic lines. For Py-GC/MS analysis, this was the only statistically significant difference for all transgenic lines. Thus, the wet chemistry and PyGC/MS results showed in agreement no distinct changes in crystalline cellulose or in the main lignin components.

**TABLE 1:** Lignin and carbohydrates contents of wood of transgenic and wild-type (WT) hybrid aspen analyzed with three methods.^a^

Line	Lignin and cellulose contents^b^									
	
	Klason lignin				Acid-soluble lignin				Cellulose
11	18.8 ± 1.1				6.0 ± 0.3				39.6 ± 2.8
14B	16.7 ± 1.1**				6.5 ± 0.4				43.7 ± 4.1
14C	18.1 ± 1.0				6.6 ± 0.6				44.3 ± 2.7
Trans	17.7 ± 1.4				6.4 ± 0.5				42.9 ± 3.8
WT	18.2 ± 0.9				6.1 ± 0.8				44.2 ± 4.1
			

**Line**	**Non-cellulosic polysaccharide composition (mol%)**^c^								
	
	**Ara**	**Rha**	**Xyl**	**Man**	**MeGlcA**	**Gal**	**GalA**	**Glc**	**GlcA**

11	2.3 ± 0.2	2.7 ± 0.1***	59.3 ± 1.7***	4.8 ± 0.1**	4.8 ± 0.4	3.5 ± 0.4**	5.6 ± 0.3	13.9 ± 0.4***	2.8 ± 0.2
14B	2.2 ± 0.1	2.7 ± 0.1*	60.1 ± 1.6***	4.3 ± 0.2	4.8 ± 0.4	2.6 ± 0.3	5.3 ± 0.2	15.4 ± 1.5***	2.7 ± 0.2
14C	2.2 ± 0.1	2.6 ± 0.1	61.7 ± 1.4	4.6 ± 0.3	5.1 ± 0.4	2.9 ± 0.4	5.3 ± 0.3	12.7 ± 1.1	2.7 ± 0.3
Trans	2.2 ± 0.1	2.7 ± 0.1***	60.4 ± 1.8***	4.5 ± 0.3	4.9 ± 0.4	2.9 ± 0.5	5.4 ± 0.3	14.1 ± 1.5***	2.7 ± 0.2
WT	2.2 ± 0.1	2.5 ± 0.1	63.2 ± 1.6	4.4 ± 0.2	4.9 ± 0.5	2.7 ± 0.7	5.2 ± 0.3	12.0 ± 0.9	2.6 ± 0.2
									

**Line**	**Py-GC/MS analysis of milled wood**^d^									
	
	**C**		**G**	**S**	**H**	**L**	**S/G ratio**		**C/L ratio**

11	74.3 ± 1.3		8.2 ± 0.5	12.1 ± 0.5	2.0 ± 0.1**	22.5 ± 1.1	1.47 ± 0.04**		3.31 ± 0.22
14B	75.6 ± 2.0		7.6 ± 0.7	11.8 ± 1.1	1.7 ± 0.2	21.3 ± 1.9	1.56 ± 0.05		3.58 ± 0.45
14C	74.4 ± 1.4		8.0 ± 0.6	12.5 ± 0.7	1.9 ± 0.2	22.6 ± 1.4	1.55 ± 0.04		3.30 ± 0.27
Trans	74.8 ± 1.7		7.9 ± 0.7	12.1 ± 0.9	1.9 ± 0.2**	22.1 ± 1.6	1.53 ± 0.06		3.41 ± 0.35
WT	74.5 ± 1.6		8.0 ± 0.7	12.5 ± 0.8	1.8 ± 0.2	22.5 ± 1.5	1.56 ± 0.08		3.33 ± 0.31

*^*a*^The analyses included two or three samples from each of the transgenic lines and eight samples from WT. Each sample consisted of a pool of two trees, and each sample was analyzed as technical triplicates. Statistical significance is based on ANOVA with *post hoc* Dunnett test for each transgenic line and on contrast for all transgenic lines (Trans) together: ^∗∗∗^*P* ≤ 0.001; ^∗∗^*P* ≤ 0.01; ^∗^*P* ≤ 0.05. ^*b*^Lignin and Updegraff cellulose content of transgenic and wild-type hybrid aspen. Data given as mass fractions in% (g/100 g dry weight). ^*c*^Monosaccharide composition of non-cellulosic polymers determined by TMS in alcohol-insoluble wood residue of transgenic and WT hybrid aspen. Data given as mol% (mol/100 mol). ^*d*^Py-GC/MS analysis of milled wood of transgenic and wild-type hybrid aspen. Data for C (carbohydrate), G (guaiacyl lignin units), S (syringyl lignin units), H (*p*-hydroxyphenyl lignin units), and L (total lignin, i.e., the sum of G, S, H, and generic phenolic constituents as defined by Gerber et al., 2012) are given as fractions in% of the total signals from the Py-GC/MS analysis. The ratio of syringyl and guaiacyl lignin units and the ratio of carbohydrate- and lignin-derived constituents are also included.*

In contrast, the monosaccharide composition did show some changes in transgenic lines compared to WT (Table 1). The xylose (Xyl) content of the transgenic lines was ∼4% lower than in the WT, whereas the glucose (Glc) and rhamnose (Rha) contents were ∼18 and ∼8% higher, respectively. These results indicate changes in the composition of the matrix polysaccharides, a decrease in xylan content, and an increase in glucan in transgenic lines.

**FIGURE 2:**
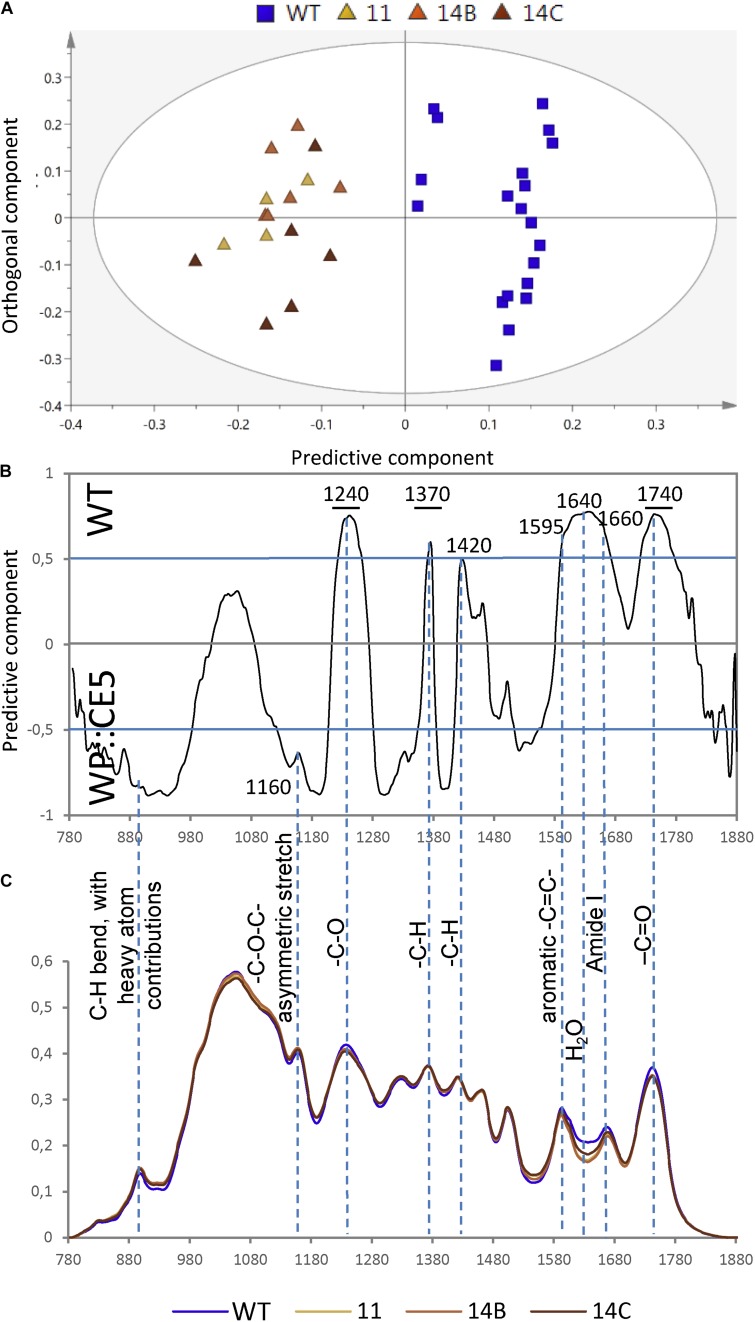
Diffuse reflectance Fourier-transform infrared (FTIR) spectra of wood of WP:CE5 transgenic (lines 11, 14B, 14C) and wild type (WT). Dotted lines show bands that are significantly different (≥50% correlation) in the transgenic lines compared to WT, according to OPLS-DA (orthogonal projections to latent structures – discriminant analysis) models using 1 + 1 (predictive + orthogonal) components. Model components are: R2X (cum) = 0.788, R2Y (cum) = 0.891, Q2 (cum) = 0.878. Score plot **(A)**, loadings **(B)** and the corresponding average spectra **(C)**.

To further reveal potential subtle chemical changes in cell walls of WP:CE5 plants, diffuse reflectance Fourier-transform infrared (FTIR) spectroscopy was applied to the ground wood samples. The spectral data analyzed by orthogonal projections to latent structures-differential analysis (OPLS-DA) showed that there was a clear separation between transgenic hybrid aspen lines and the WT (Figure 2A). The bands at 1240, 1370, and 1740 cm^–1^, which originate from C-O stretching, CH_2_ bending and C = O stretching vibrations (Gorzsas et al., 2011), respectively, all present in acetyl ester groups, strongly contributed to this separation and these signals were less abundant in the transgenic plants than in the WT (Figures 2B,C). This result was similar to that obtained with the previously studied transgenic plants overexpressing *AnAXE1* (Pawar et al., 2016, 2017a). On the other hand, the band at 1600 cm^–1^, reflecting aromatic C = C vibrations found abundantly in lignin, and the region around 1640 cm^–1^, reflecting water, were more abundant in WT plants, which was in contrast to results observed with *AnAXE1*-expressing *Arabidopsis* and aspen (Pawar et al., 2016, 2017a). The latter results suggest some changes in lignin structure and cell wall hydration that are specific to CE5 overexpressors.

### Xylan Acetylation

**FIGURE 3:**
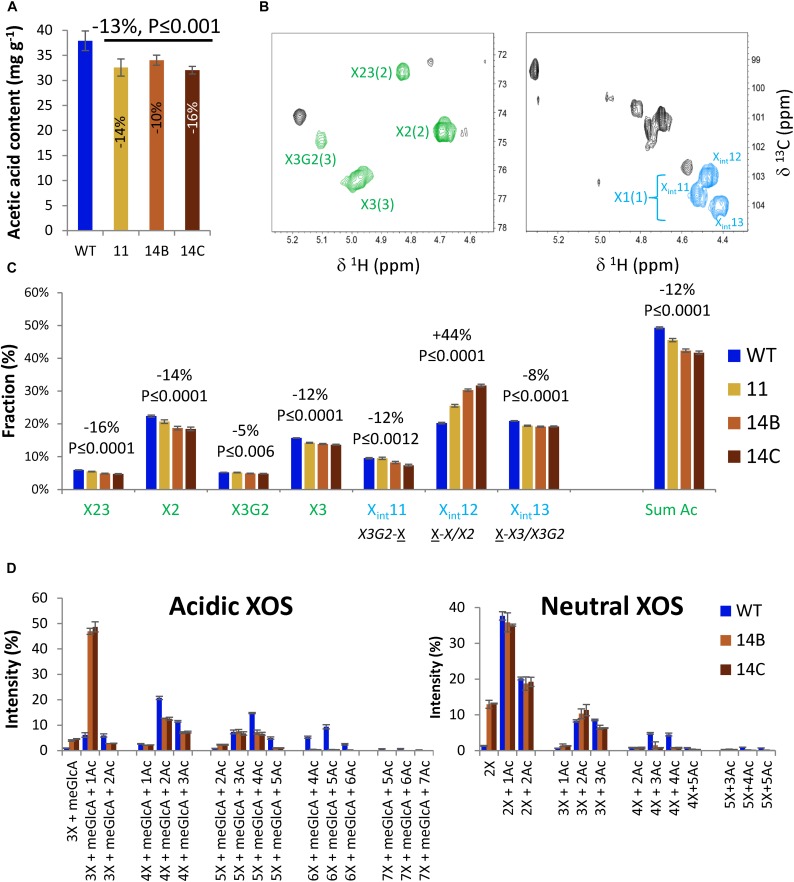
Effects of WP:CE5 expression (lines 11, 14B, and 14C) on xylan acetylation in aspen wood. **(A)** Cell wall acetyl content determined by acetic acid release. **(B)** 2D qHSQC NMR spectra of extracted xylan showing signals from acetylated (green) and non-acetylated (blue) Xyl*p* units in xylan, which were used for quantification in **(C)**. X23-diacetylated Xyl*p*; X2-Xyl*p* monoacetylated at position 2; X3G2-Xyl*p* acetylated at position 3 and glucuronosylated at position 2; numbers in parenthesis correspond to carbon number in Xyl*p;* Xint – different internal Xyl*p* signals as assigned by Grantham et al. (2017). **(D)** OLIMP analysis of acidic and neutral xylo-oligosaccharides (XOS) released by endoxylanase *Aa*GH10. Data are means ± *SE*, *N* = 2 or more biological × 3 technical replicates for **(A)**, 2 or more biological × 2 technical replicates for **(C)**, and 2 biological replicates for **(D)**. *P*-values in **(A,C)** correspond to post-ANOVA contrast analysis comparing all transgenic lines to WT.

Quantitation of acetic acid released through saponification of wood showed that the transgenic plants had reduced acetyl content by 10 to 16%, compared to the WT (Figure 3A).

The acetylation was further investigated using 2D HSQC NMR spectroscopic analysis of DMSO-extracted xylan (Supplementary Data Sheets S2, S3), which revealed the presence of acetylated and non-acetylated Xyl*p* residues (Figure 3B). The signals shown in green were used to obtain the relative content of acetylated Xyl*p* units whereas the signals shown in blue represent different non-acetylated Xyl*p*. For the WT, 49% of the total Xyl*p* units were acetylated. That included 22% monoacetylation at position C-2 (X2), 16% monoacetylation at position C-3 (X3), 6% di-acetylation at positions C-2 and C-3 (X23), and 5% acetylation at C-3 and meGlcA (X3G2) (Figure 3C). Transgenic lines exhibited reductions in signals from all acetylated Xyl*p*, by 16% for X23, 14% for X2, 5% for X3G2, and 11% for X3. In total, the content of acetylated Xyl*p* units was reduced by 12% in transgenic lines compared to the WT (Figure 3C). Moreover, there was a 44% increase in the content of non-acetylated Xyl*p* units preceeding either non-acetylated or C-2 acetylated units (X-X/X2). The results suggest that the CE5 enzyme acted on Xyl*p* positions 2 and 3, and could also deacetylate 2,3-double-acetylated Xyl*p* as well as position 3 in glucuronosylated Xyl*p* units of aspen wood xylan.

Moreover, to investigate the changes in the pattern of glucuronoxylan acetylation in the transgenic plants, heat-treated AIR samples from lines 14A and 14B and from the WT were treated with the *Aa*GH10 endo-1,4-β-xylanase, and the released xylo-oligosaccharides (XOS) were analyzed by using oligosaccharide mass profiling (by AP-MALDI-ITMS; Chong et al., 2011). The treatment released acidic XOS with a degree of polymerization (DP) of three to seven and with up to seven acetyl groups (Figure 3D). The transgenic lines showed a prominent change in the distribution of acidic XOS compared to the WT. Whereas the most abundant acidic XOS in the transgenic lines had a DP of three and one acetyl group, the most abundant acidic XOS in the WT had a DP of four and two acetyl groups, although XOS with a DP of five with four acetyl groups and XOS with a DP of six with four or five acetyl groups were also common. In comparison with the WT, the distribution of acidic XOS products with a DP of four and five exhibited a shift toward less acetylated products for the transgenic plants. Furthermore, only the WT yielded noticeable quantities of acidic XOS with a DP of six or seven. This indicates that the glucuronoxylan of the transgenic plants was more accessible to the xylanase treatment and that the main products were less acetylated. The neutral XOS had a DP of two to five and up to five acetyl groups in both genotypes, but the transgenic lines had more DP 2 products without acetyl groups compared to WT (Figure 3D). The results indicate that the CE5 enzyme caused reduced acetylation of xylan for both neutral and meGlcA-substituted domains in glucuronoxylan.

### Saccharification

**FIGURE 4:**
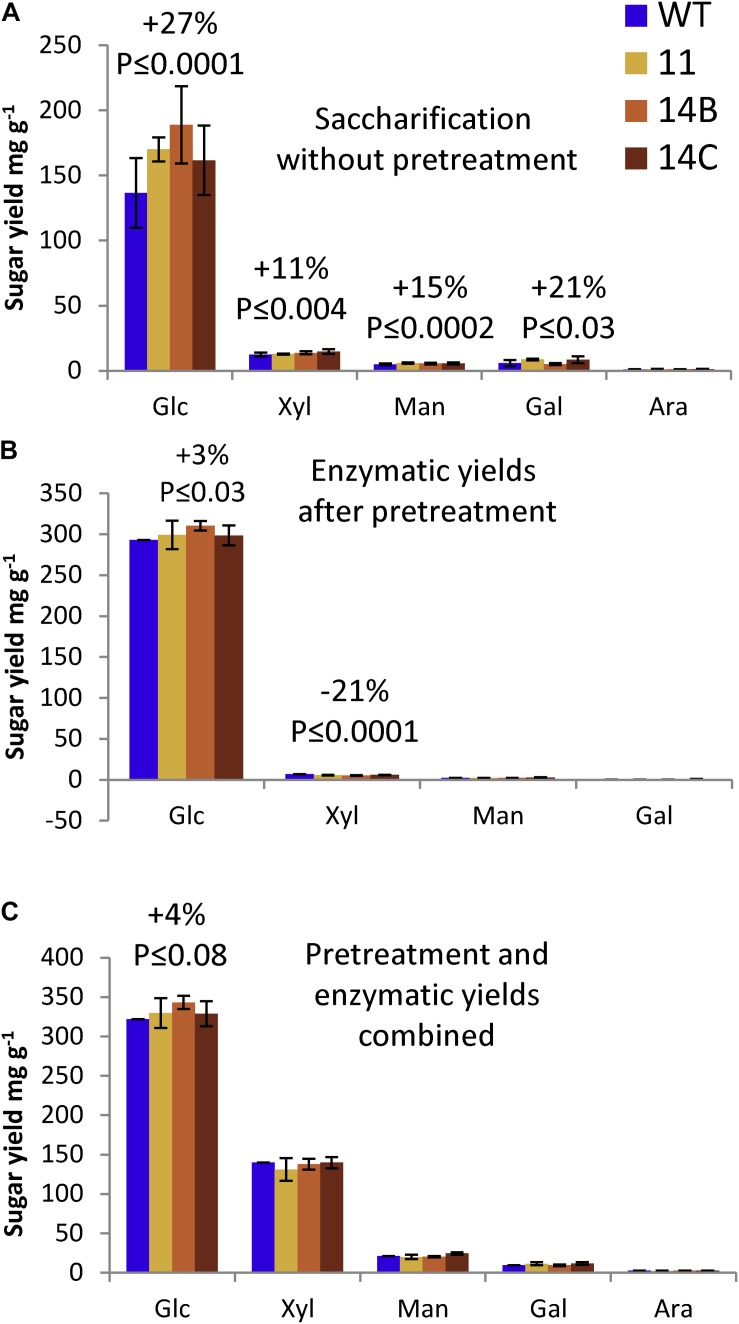
Effects of WP:CE5 expression on saccharification. **(A)** Sugar yields of enzymatic hydrolysis without pretreatment. **(B)** Sugar yields of enzymatic hydrolysis after acid pretreatment. **(C)** Combined sugar yields of pretreatment liquid and enzymatic hydrolyzate. Data are means ± *SE*, *N* = 3 technical replicates of a pooled material from four trees. *P*-values correspond to ANOVA contrast analysis comparing all transgenic lines to WT.

In the screening of the original 11 transgenic lines, the three lines (11, 14B, and 14C) showing the highest transcript levels for the transgene were also the only lines that gave improved glucose yield in enzymatic saccharification of non-pretreated wood, suggesting that a threshold of transgene expression was needed to achieve measurable saccharification gains. Analytical enzymatic saccharification of non-pretreated wood (Figure 4A) resulted in a glucose yield of 137 mg/g for the WT (34.1% glucan recovery based on Updegraff cellulose). For the transgenic lines (average for 11, 14B, and 14C) there was a 27% improvement in glucose yield compared to the WT (glucose yield: 174 mg/g; glucan recovery: 44.6%). Although the transgenic lines contained similar fractions of mannose and galactose as the WT and although they had 5% lower content of xylose, enzymatic saccharification of non-pretreated transgenic lines resulted in higher yields of xylose, mannose, and galactose compared to the WT (11–21% improvement, Figure 4A). Glucose production rate analysis after 2 h of enzymatic hydrolysis also pointed toward higher rates of glucose release for transgenic plants (Supplementary Figure S1). The results show that expression of CE5 AXE improved the digestibility of non-pretreated transgenic aspen with regard to both cellulose and hemicellulose.

With acid pretreatment, the transgenic aspen gave 8% higher glucose yield in the pretreatment liquid than the WT (data not shown), and also 3% higher glucose yield in the enzymatic saccharification (Figure 4B). Most of the xylan was hydrolyzed in the acid pretreatment (data not shown), which resulted in higher xylan recoveries for the transgenic aspen (59.1%) than for the WT (55.6%) even though the total xylose yield was not affected (Figure 4C). When the sugars released in both the pretreatment liquid and the enzymatic hydrolyzate are combined, the WT reached 65.7% glucan recovery and 58.6% xylan recovery, whereas the transgenic aspen reached 70.2% glucan recovery and 61.7% xylan recovery. The results show that the transgenic aspen had improved glucan recovery and yields, and indicate that the xylan in the transgenic aspen was easier to degrade during the pretreatment.

### Wood Nanostructure

Two methods were employed to compare the properties of the cell wall nanostructure of the transgenic plants and the WT, viz. Brunauer–Emmett–Teller (BET) analysis (Brunauer et al., 1938) and Simons’ staining (Chandra and Saddler, 2012). While BET analysis was carried out using air-dried wood powder or pretreated wood powder, Simons’ staining was carried out using wood powder or pretreated wood powder suspended in water to simulate the conditions during a saccharification reaction.

BET analysis, which measures physical adsorption of nitrogen gas to the solid phase, provided information about the specific surface area (Table 2). On an average, both non-pretreated and pretreated wood of the transgenic plants exhibited larger surface area than the WT. The average surface area was 11% larger for the transgenic plants than for the WT for both materials. The increase of the surface area after pretreatment agrees with the fact that the susceptibility to enzymatic saccharification also increased after pretreatment.

**TABLE 2:** BET surface area (m^2^/g) of wood of transgenic and wild-type (WT) hybrid aspen with and without acid pretreatment.^a,b^

Wood samples	Non-pretreated wood	Acid-pretreated wood
11	2.06 ± 0.04***	3.22 ± 0.09
14B	1.91 ± 0.13*	3.29 ± 0.26*
14C	1.75 ± 0.07	3.33 ± 0.08**
Trans	1.91 ± 0.15***	3.28 ± 0.15**
WT	1.72 ± 0.07	2.96 ± 0.02

*^a^Analyses were based on samples consisting of pooled wood from four separate trees. Each sample was analyzed as technical triplicates. ^b^Statistical significance is based on ANOVA with *post hoc* Dunnett test for each transgenic line and on contrast for all transgenic lines (Trans) together: ^∗∗∗^*P* ≤ 0.01; ^∗∗^*P* ≤ 0.05; ^∗^*P* ≤ 0.1.*

**FIGURE 5:**
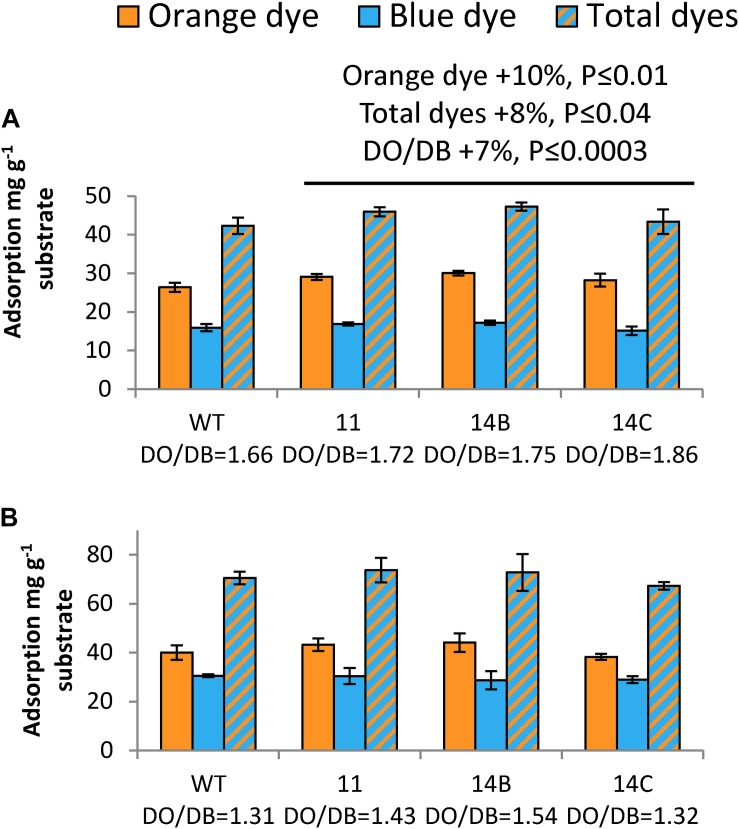
Effects of WP:CE5 expression on wood nanostructure. Simons’ staining of non-pretreated **(A)** and acid-pretreated aspen wood **(B)**. Data are means ± *SE*, *N* = 3 technical replicates. *P*-values correspond to ANOVA contrast analysis comparing all transgenic lines to WT.

The Simons’ stain assay (Figure 5) is based on two dyes: blue (DB), which has a molecular mass of 993 Da, and orange (DO), which is filtered to obtain its high-molecular-mass (>100 kDa) fraction (Yu et al., 1995). Using these dyes, the accessible surface area and the porosity of the samples can be evaluated. For non-pretreated wood, transgenic plants exhibited 10% higher adsorption of DO than the WT, and 8% higher adsorption of total dyes (Figure 5A). However, this difference between the transgenic plants and the WT disappeared after pretreatment (Figure 5B). Comparing the adsorption ratio DO/DB, the transgenic plants showed 7% higher values than the WT without pretreatment. All samples showed a decreased DO/DB ratio after pretreatment, as the pretreatment resulted in increased adsorption of DB.

Data from both BET and Simons’ staining agree that transgenic plants had an advantage over the WT with regard to structural properties, such as larger surface area and better accessibility. In summary, the activity of the CE5 AXE enlarged the surface area of the transgenic aspen and improved its accessible surface area, which was especially evident for non-pretreated wood.

## Discussion

Biomass recalcitrance to enzymatic saccharification is one of the main problems that need to be solved in order to implement technologies delivering green chemicals and fuels from renewable plant biomass. Since reducing xylan acetylation was suggested as one of the most promising strategies for reducing lignocellulose recalcitrance and increasing bioethanol yields (Donev et al., 2018), we studied the effectiveness of *in planta* expression of an AXE from family CE5 to reduce biomass acetylation. Here we present analyses of transgenic aspen expressing *Hj*AXE driven by a wood-specific promoter, and targeted to cell walls using a plant signal peptide. *Hj*AXE represents a distinct AXE belonging to a different CE family than the previously used *An*AXE1 from CE1 (Pawar et al., 2016, 2017a). Although both these enzymes were active on polymeric xylan, there were subtle differences in their activities *in vitro* (Koutaniemi et al., 2013), and substantial differences with respect to their amino-acid sequences, the three-dimensional structure of their catalytic sites, and their domain structures (Margolles-Clark et al., 1996; Hakulinen et al., 2000).

### CE5 AXE Can Efficiently Deacetylate Cell Wall Xylan *in vivo*

The transgenic plants expressing the CE5 member *Hj*AXE exhibited decreased xylan acetylation in their wood by 10–16% compared to the WT (Figure 3A), which is comparable with the reductions observed in previously studied plants expressing the CE1 enzyme *An*AXE1 (Pawar et al., 2016, 2017a) or plants with suppressed RWA gene expression (Pawar et al., 2017b). While these reductions were relatively modest, they had significant impact on xylan digestibility by endoxylanases. Overexpression of *Hj*AXE prominently affected the distribution of acidic xylo-oligosaccharides released by endoxylanase *Aa*GH10 (Figure 3D). As in *An*AXE1-expressing plants and RWA-suppressed plants, the 2D qHSQC NMR signals from *Hj*AXE-expressing plants indicated that Xyl*p* units were deacetylated at both C-2 and C-3 positions of Xyl*p* (Figures 3B,C). This is consistent with previous reports on deacetylation by *Hj*AXE *in vitro* where activity at positions 2 and 3 was detected, and a preference for monoacetylated Xyl*p* (Biely et al., 1997; Hakulinen et al., 2000; Biely et al., 2011; Neumüller et al., 2015). It was suggested that the deacetylation of glucuronosylated Xyl*p* would be difficult based on the structure of the catalytic site (Hakulinen et al., 2000). In agreement, no such activity was observed *in vitro* when acetylated oligosaccharides were used as substrates (Neumüller et al., 2015). However, a significant decrease in X3G2 units accompanied by significant decrease in internal Xylp preceeded by X3G2 (X3G2-X) in WP:CE5 (Figure 3C), which was not observed in any of the previously studied transgenic plants, suggested a possibility of deacetylation at C-3 position in the glucuronosylated Xyl*p* units by *Hj*AXE. This conclusion is in line with the observed increase in completely deacetylated 3X + MeGlc xylo-oligosaccharides observed in WP:CE5 lines (Figure 3D), which would be expected assuming that *Hj*AXE could deacetylate glucuronosylated Xyl*p*. While this is a novel observation, these results might reflect endogenous GH10 activity (Derba-Maceluch et al., 2015), which creates a free C-4 position at the non-reducing end enabling the migration of an acetyl group to this position (Mastihubova and Biely, 2004). However, we cannot exclude that *Hj*AXE could exhibit additional specificities when acting on native xylan associated with cellulose microfibrils, especially that their cellulose-binding domain could precondition them on cellulose-bound substrates.

### HjAXE Does Not Impair Plant Growth

Transgenic plants expressing *Hj*AXE exhibited good height and diameter growth in the greenhouse during a 2-month cultivation period. The only difference from WT was a shorter internode length, which indicates an increased leaf production. It would need to be further studied if such an increase could result in more biomass production by the transgenic lines, and if the field performance of these lines is satisfactory.

### HjAXE Targeted to Secondary Cell Walls Improves Woody Biomass Saccharification

Xylan of transgenic plants was shown to be more accessible to hydrolysis by *Aa*GH10 xylanase (Figure 3D), confirming previous observations of synergy between AXEs and xylanases in xylan hydrolysis (Biely et al., 1986). Interestingly, the WP:CE5 lines had reduced Xyl content in matrix polysaccharides (Table 1), indicative of increased hydrolysis of the deacetylated xylan *in muro* by cell-wall-residing native GH10 enzymes, xylanases and/or transglycosylases (Derba-Maceluch et al., 2015). Similar reductions were observed in other transgenic lines with reduced xylan acetylation (Pawar et al., 2017a, b). If cell-wall-residing xylan was indeed partially hydrolyzed by plant GH10 enzymes, then increased cell-wall porosity, and increased accessibility to cellulose leading to increased glucose yields would be expected. Our analyses demonstrate both.

Increased surface area and porosity of lignocellulose from transgenic plants with reduced acetylation was for the first time demonstrated in this study using BET analysis and Simons’ staining (Table 2 and Figure 5). We speculate that increased surface area and porosity is a direct consequence of removal of cell-wall-residing xylan by endogenous GH10 enzymes in the cell walls of transgenic plants. Interestingly, the decrease in xylan biosynthesis that reduces xylan content is also known to improve saccharification but only when coupled with reduction in xylan chain length (Lee et al., 2011a; Ratke et al., 2018).

A 27% increase in glucose yield and 11% increase in xylose yield in enzymatic saccharification of non-pretreated wood, as well as a 3% increase in glucose yield after acid pretreatment and enzymatic saccharification were observed in WP:CE5 lines (Figure 4). Similarly increased sugar yield of enzymatic saccharification without pretreatment was reported for transgenic aspen and *Arabidopsis* with reduced xylan acetylation by either supressing native RWA genes or by overexpressing CE1 AXE (Pawar et al., 2016, 2017a,b). Similarly, the positive influence was smaller after acid pretreatment (Pawar et al., 2017a, b). The observed reductions in recalcitrance could be a direct consequence of increased porosity and accessibility due to xylan deacetylation by CE5 AXE acting in close proximity to cellulose.

Moreover, altered xylan acetylation is thought to highly affect cell wall architecture since the acetylation pattern was shown to mediate xylan binding to cellulose microfibrils (Grantham et al., 2017) as well as xylan covalent linkages to lignin (Giummarella and Lawoko, 2016). This could be the basis of the apparently paradoxical observation that increased acetylation in mutant rice (Zhang et al., 2017) and in transgenic poplar (Yang et al., 2017) resulted in improved saccharification. Applying tools to probe cell wall porosity, and cellulose-xylan as well as lignin-xylan interactions in these acetylation-altered plants could possibly give more definite explanations regarding their recalcitrance behavior.

## Conclusion

Expression *in planta* of *Hj*AXE from family CE5 leads to reduced xylan acetylation and approximately 30% increased glucose yields in enzymatic saccharification of wood without pretreatment, as well as 3% improved glucose yields even when using industrially relevant pretreatment conditions. Plants expressing *Hj*AXE show good growth, and similar improvement of saccharification and reduction in xylan content as the previously studied plants expressing *An*AXE1 from family CE1, although the cell wall chemotypes and de-acetylation patters show subtle differences between these two types of transgenics. Increased cell wall nanoporosity likely plays a key role in reducing the recalcitrance by *Hj*AXE expression. Structural analyses based on BET and Simons’ staining emerge as useful tools for understanding differences in recalcitrance of engineered transgenic wood.

## Data Availability Statement

All datasets generated for this study are included in the article/Supplementary Material.

## Author Contributions

PP and MD-M prepared plant material, analyzed transcript levels, and determined acetyl esterase activity. ZW and PP performed compositional analysis. MH performed NMR analyses. S-LC performed OLIMP analyses. ZW performed pretreatment and enzymatic saccharification, BET analysis, and Simons’ staining. EM and ZW performed statistical analyses. EM, LJ, and MT conceived the study and supervised the experiments. ZW, LJ, and EM wrote the manuscript with contributions from all authors.

## Conflict of Interest

The authors declare that the research was conducted in the absence of any commercial or financial relationships that could be construed as a potential conflict of interest.

## Footnotes

1 www.jmp.com

## Supplementary Material

DATA SHET S1 Table S1 and Figure S1.

DATA SHEET S2 NMR Data Part 1.

DATA SHEET S3 NMR Data Part 2.
