# Supplementary material for: Hybrid Aspen Expressing a Carbohydrate Esterase Family 5 Acetyl Xylan Esterase Under Control of a Wood-Specific Promoter Shows Improved Saccharification
Source: Front Plant Sci. 2020 Apr 8;11:380. doi: 10.3389/fpls.2020.00380 (PMC7156598; doi:10.3389/fpls.2020.00380)
Supplement: DATA SHET S1 — Table S1 and Figure S1. [file Data_Sheet_1.PDF]

SUPPLEMENTARY MATERIAL

Supplementary Table S1. Primers used for qPCR analysis.

Primer name	Primer sequence	Length of amplicon (bp)	Efficiency	Gene ID
TUB_for	ATTCCCTCGCCTTCATTTCT	200	1.9	Potri.001G464400
TUB_rev	CCTCTTTCGTGCTCATCTTACC			
UBQ-L_for	TGGCAAGACCATAACTCTCG	200	1.86	Potri.005G198700
UBQ-L_rev	CTCCCCTAAGCCTCAAAACC			
CE5 for1	GCTGTTTCTGCTGTGAAGGC	202	1.91	*Hj*AXE
CE5 rev2	GGTGAACGTTTGGATCATTTCC			

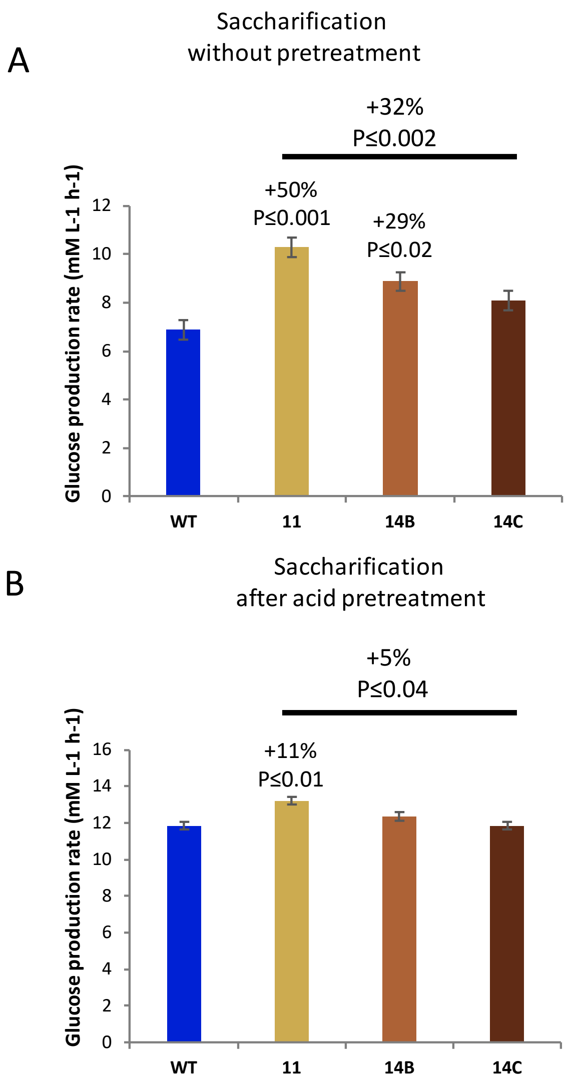

Supplementary Figure S1. Glucose production rate (GPR) after 2 h of enzymatic hydrolysis: (A) without pretreatment; (B) with acid pretreatment. Data are means ± SE, N=3 technical replicates of samples of pooled trees. P values correspond to post-hoc Dunnett test for individual lines, and ANOVA contrast analysis comparing all transgenic lines to WT.
