# Supplementary figures and images for: Hybrid Aspen Expressing a Carbohydrate Esterase Family 5 Acetyl Xylan Esterase Under Control of a Wood-Specific Promoter Shows Improved Saccharification
Source: Front Plant Sci. 2020 Apr 8;11:380. doi: 10.3389/fpls.2020.00380 (PMC7156598; doi:10.3389/fpls.2020.00380)

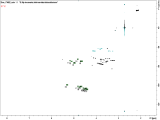

Supplement: DATA SHEET S2 — NMR Data Part 1. [file Data_Sheet_2.ZIP › Wang_NMR data/1/pdata/1/thumb.png]

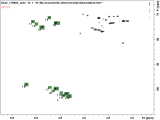

Supplement: DATA SHEET S2 — NMR Data Part 1. [file Data_Sheet_2.ZIP › Wang_NMR data/10/pdata/1/thumb.png]

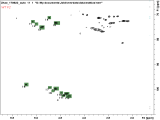

Supplement: DATA SHEET S2 — NMR Data Part 1. [file Data_Sheet_2.ZIP › Wang_NMR data/11/pdata/1/thumb.png]

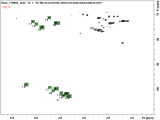

Supplement: DATA SHEET S2 — NMR Data Part 1. [file Data_Sheet_2.ZIP › Wang_NMR data/12/pdata/1/thumb.png]

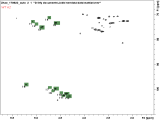

Supplement: DATA SHEET S2 — NMR Data Part 1. [file Data_Sheet_2.ZIP › Wang_NMR data/2/pdata/1/thumb.png]

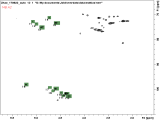

Supplement: DATA SHEET S3 — NMR Data Part 2. [file Data_Sheet_3.ZIP › Wang_NMR data/13/pdata/1/thumb.png]

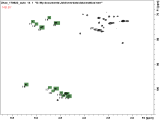

Supplement: DATA SHEET S3 — NMR Data Part 2. [file Data_Sheet_3.ZIP › Wang_NMR data/14/pdata/1/thumb.png]

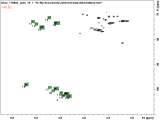

Supplement: DATA SHEET S3 — NMR Data Part 2. [file Data_Sheet_3.ZIP › Wang_NMR data/15/pdata/1/thumb.png]

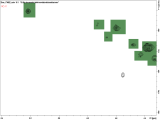

Supplement: DATA SHEET S3 — NMR Data Part 2. [file Data_Sheet_3.ZIP › Wang_NMR data/16/pdata/1/thumb.png]

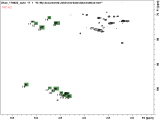

Supplement: DATA SHEET S3 — NMR Data Part 2. [file Data_Sheet_3.ZIP › Wang_NMR data/17/pdata/1/thumb.png]
